# Supplementary material for: Effect of Vancomycin, Gentamicin and Clindamycin on Cartilage Cells In Vitro
Source: Biomedicines. 2023 Nov 25;11(12):3143. doi: 10.3390/biomedicines11123143 (PMC10740484; doi:10.3390/biomedicines11123143)
Supplement: Supplementary file 1 [file biomedicines-11-03143-s001.zip › biomedicines-2657408-supplementary.pdf]

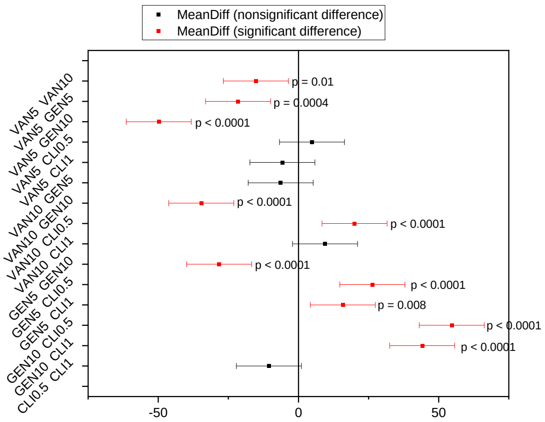

48 h

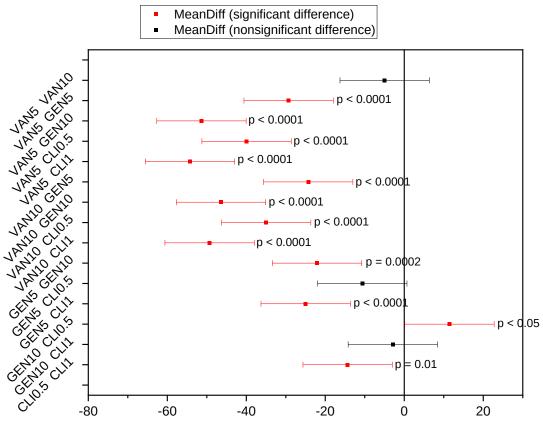

72 h

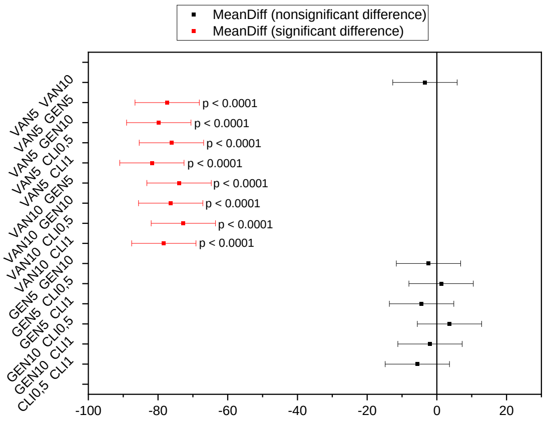

168 h

Figure S 1: Significances in cytotoxicity after 48, 72 and 168 hours; VAN (Vancomycin); CLI (Clindamycin); GEN (Gentamicin); significant difference = blue; non-significant difference = black).
